# Quality Control of Human Pluripotent Stem Cell Colonies by Computational Image Analysis Using Convolutional Neural Networks

**DOI:** 10.3390/ijms24010140

**Published:** 2022-12-21

**Authors:** Anastasiya Mamaeva, Olga Krasnova, Irina Khvorova, Konstantin Kozlov, Vitaly Gursky, Maria Samsonova, Olga Tikhonova, Irina Neganova

**Affiliations:** 1 Mathematical Biology and Bioinformatics Lab, Peter the Great St. Petersburg Polytechnic University, 195251 Saint Petersburg, Russia; 2 Institute of Cytology, 194064 Saint Petersburg, Russia; 3 Faculty of Biology, Saint-Petersburg State University, 199034 Saint Petersburg, Russia; 4 Ioffe Institute, 194021 Saint Petersburg, Russia; 5 Institute of Biomedical Chemistry, 119121 Moscow, Russia

**Keywords:** human pluripotent stem cells, pluripotency, deep learning, convolutional neural networks, image processing

## Abstract

Human pluripotent stem cells are promising for a wide range of research and therapeutic purposes. Their maintenance in culture requires the deep control of their pluripotent and clonal status. A non-invasive method for such control involves day-to-day observation of the morphological changes, along with imaging colonies, with the subsequent automatic assessment of colony phenotype using image analysis by machine learning methods. We developed a classifier using a convolutional neural network and applied it to discriminate between images of human embryonic stem cell (hESC) colonies with “good” and “bad” morphological phenotypes associated with a high and low potential for pluripotency and clonality maintenance, respectively. The training dataset included the phase-contrast images of hESC line H9, in which the morphological phenotype of each colony was assessed through visual analysis. The classifier showed a high level of accuracy (89%) in phenotype prediction. By training the classifier on cropped images of various sizes, we showed that the spatial scale of ~144 μm was the most informative in terms of classification quality, which was an intermediate size between the characteristic diameters of a single cell (~15 μm) and the entire colony (~540 μm). We additionally performed a proteomic analysis of several H9 cell samples used in the computational analysis and showed that cells of different phenotypes differentiated at the molecular level. Our results indicated that the proposed approach could be used as an effective method of non-invasive automated analysis to identify undesirable developmental anomalies during the propagation of pluripotent stem cells.

## 1. Introduction

Human pluripotent stem cells (hPSCs) are of extensive use in modern regenerative medicine due to their properties of unlimited self-renewal and the ability to differentiate into all cell types of the human body [1]. By applying specific reprogramming factors to human somatic cells, human induced pluripotent stem cells (hiPSCs) can be generated [2], providing a promising instrument for the patient-specific treatment of multiple diseases [3,4]. However, the efficient translation of hiPSCs requires scalable cell manufacturing strategies for optimal self-renewal and functional differentiation. Traditional manual cell culture is variable and labor intensive, posing challenges for high-throughput applications. The manual maintenance of hPSCs introduces several limitations for its transition into large-scale experiments. First, the maintenance of the hPSC culture requires highly trained and experienced staff. Technician variability and human error pose major limitations when high numbers of samples are being processed in parallel. This variability also contributes to significant differences between the cell lines generated in various laboratories [5,6].

Thus, there is an increasing need for reproducible large-scale stem cell and differentiated progeny production, with minimal variation, rendering manual approaches impracticable. Overcoming these limitations by moving toward an automated process will allow the handling of a greater numbers of cells, which will in turn facilitate the optimization of the existing protocols for cell maintenance and directed differentiation. The use of fully automated platforms for hiPSC derivation, expansion, and differentiation may be key in transitioning to large-scale cell culture [7,8,9,10].

Recently, using cell image analysis of the hPSCs, we demonstrated that classification based on the various morphological parameters characterizing the size and shape of both hESC and hiPSC colonies could reliably predict the pluripotency potential of the colonies from three hPSC lines [11]. Currently, hPSC colony morphology is considered as an important criterion of their pluripotent state and ability to self-renewal. Recently, we analyzed seven morphological parameters of hESC line H9 and three hiPSC lines divided by their morphological appearance on colonies with good and bad phenotype and confirmed our morphological examination by qRT-PCR of the 14 pluripotency markers’ gene expression, along with the competence to differentiate into three germ layers via the embryoid body differentiation protocol. Employing the analysis of variance for the morphological parameters, we demonstrated that the selected morphological parameters carried information concerning the different cell lines and different phenotypes within each line. As such, we demonstrated that a model of the classification of colonies by phenotype, built on the selected morphological parameters as predictors, recognized the phenotype with an accuracy of 70–75%. This allows us to use the same approach for the morphological evaluation of the phenotype as either good or bad in the present study.

The computational tools for biological image analysis are extensively used for the automated assessment of cellular morphology [12,13]. One general approach in such analysis consists of extracting various features (descriptors) from raw images and selecting the most informative features for classification or regression tasks [14,15,16]. A multi-purpose image classifier Wndchrm and an open-source utility based on this algorithm were developed and shown to be useful for a wide range of classification problems in biological image analysis [15,17]. A Wndchrm-based classifier was used to distinguish pluripotent hiPSCs from improperly reprogrammed cells and to show that nuclear subdomains were the most informative for this discrimination [18]. A combination of automated live-cell imaging and algorithms for colony morphology analysis were applied to quantitatively evaluate the definition of hPSC colony morphology as an important criterion of its healthy state [19]. A computational system for the time-lapse imaging analysis was developed, which implemented a machine learning-based classification, segmentation, and statistical modelling to determine hiPSC colony formation and predict the best hiPSC selection phase [20].

Another approach to image analysis involves the application of deep learning methods [21]. Convolutional neural networks (CNNs) have become the leading classification systems in visual recognition, including the classification problems related with cell images [13,22,23,24,25,26]. It was shown that CNN-based classification for various cell types was more effective than other methods, including the aforementioned Wndchrm [27]. CNNs do not require a priori feature extraction from the input image, but instead transform the image to a multi-level representation using a series of convolutional layers, with higher layers providing a more abstract representation. When a CNN-based model is trained for classification, the information from higher layers is tuned to increase the properties of the input image that are important for distinction and to reduce non-informative variations. Therefore, CNNs extract image features (“feature maps”) in an automated fashion, which allows their application to images after basic preprocessing or without preprocessing at all.

Despite the variety of proposed automated alternatives, a common non-invasive approach for detecting morphological changes in hPSC colonies associated with the loss of pluripotency and clonality relies heavily on the researcher’s experiences. In this study, we tried to solve a practical problem related to the automation of this approach, which would reduce the inevitable errors due to the human factor and make the colony selection more reliable during multiple passaging. We developed a CNN-based classifier of the colony status and trained it on hESC line H9 data, phenotyped by an expert. Instead of the tedious extraction and analysis of morphological parameters with a subsequent analysis, we investigated whether it was possible to translate the expert knowledge to a reliable decision about colony phenotype using a deep-learning analysis of input images without segmentation. We also found a specific spatial scale in the image that was the most informative in the context of this knowledge.

The spontaneous differentiation of hPSCs leads to changes in both the cellular phenotype and long-term colony morphology, which can also be observed in the proteomic landscape, i.e., the colony phenotype can be linked to specific changes in the expression of various proteins, not necessarily known as pluripotent markers [28]. As an additional mode of analysis, we examined the proteomic data from several H9 cell samples with different phenotypes. We showed that cells with different phenotypes are associated with differently expressed proteins.

## 2. Results

### 2.1. Image Acquisition and Phenotyping

**Figure 1:**
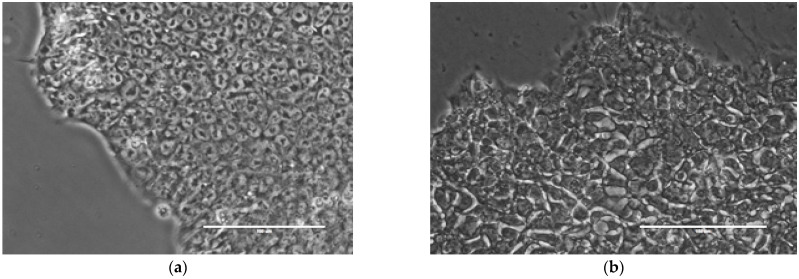
Examples of hPSC colonies with (**a**) good and (**b**) bad phenotypes. Criteria for visual assessment of morphological features associated with the phenotype are given elsewhere [11]. Scale bar, 100 µm. More examples are shown in Figure S1.

We collected 269 phase-contrast images of hPSC colonies from the H9 line. All of the colonies were phenotyped as “good” or “bad” through visual analysis, according to their morphological properties, which are associated with either a high or low pluripotency status, respectively. Figure 1 shows examples of the colony images from the two classes. The full dataset contained 137 (50.9%) images of good and 132 (49.1%) images of bad colonies.

### 2.2. CNN-Based Automated Classification of hPSC Colonies according to Their Phenotype

As a tool for the automated classification of hPSC colony images into either a good or bad phenotype class, we implemented a CNN model with a configuration proposed by the Visual Geometry Group (VGG), University of Oxford [29], which demonstrated the best performance in previous studies of classification tasks for several cell types [27]. We split the dataset into the training and validation parts and trained the model under different conditions, i.e., for various specific forms of the VGG network architecture, different data processing methods and different image augmentation means are required.

First, we investigated the performance of the VGG network with different architectures (see Table S1 for more details of the architectures). VGG13 showed the best classification quality on the validation set according to most quality measures (Table 1), predicting good and bad colonies with 83% accuracy. Therefore, we selected this network for further study.

Next, we analyzed how different image processing methods affected the model performance. As CNNs are designed to automatically extract low-to-high level features from images and effectively filter irrelevant variations, their performance should exhibit low sensitivity to image preprocessing. We considered the following four image preprocessing methods: gray level transformation, intensity normalization, binarization, and normalization using histogram equalization. To analyze how preprocessing could influence the model performance, we applied each method separately to the input images and retrained the VGG13 model on the preprocessed data. The classification appeared to be more effective with the histogram equalization applied to the input images (Table 2). Therefore, we fixed this data preprocessing method for further study.

Deep learning models are most effective on large training datasets. Smaller sets are usually increased using augmentation, which artificially creates new input images by applying various geometric transformations. Augmented images also add a new source of irrelevant variation to the training dataset, thus helping the model to recognize it. We considered the following augmentation methods: random cropping, random rotation (including image transposition and flipping), and a combination of cropping and rotation. To analyze how augmentation could influence the model performance, we applied each method separately to the input images, which were preprocessed with histogram equalization, and retrained the VGG13 model on the modified dataset. The classification appeared to be more effective when both augmentation methods were applied to the input images (Table 3).

As a result, we found the best combination of preprocessing, augmentation, and CNN configuration that led to the model that classified the hPSC colonies from the validation set according to their phenotype with the 89% accuracy (Table 3 and Table 4).

### 2.3. Characteristic Spatial Scale for Assessing the Morphological Phenotype

The morphological phenotype of the hPSC colony associated with pluripotency is determined by the morphological parameters that characterize both the cells inhabiting this colony and the colony as a whole [11]. These parameters cover spatial scales ranging between the typical size of a cell (~10–15 µm) and the typical size of a mature colony (~500 µm). As we constructed an automated classifier that could recognize the phenotype with good accuracy, we aimed to find a spatial scale that was the most informative in the learning process. To this end, we trained a classifier on images of different sizes and determined the size that was associated with the best model performance.

**Figure 2:**
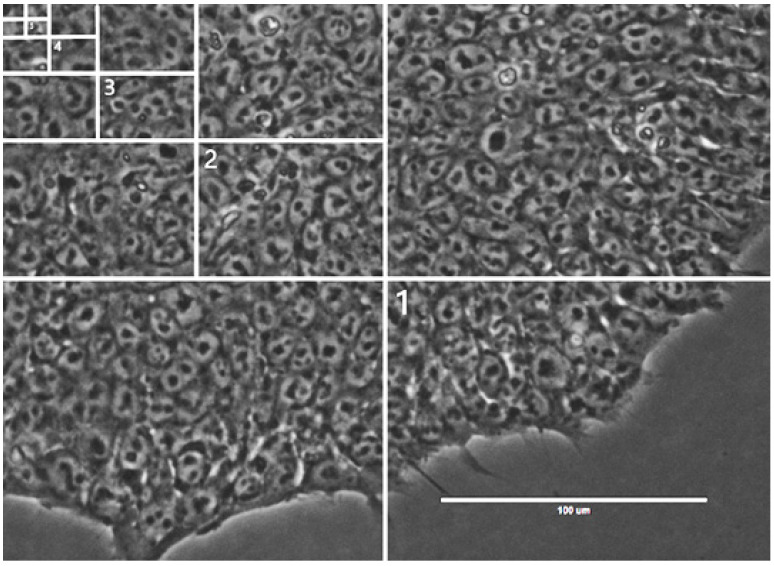
Illustration of the process of sequentially cutting an image into smaller pieces. The numbers in the figure indicate examples of images obtained at each step of this process.

We cut each image from our initial dataset into four equal parts and used the resulting smaller images as a new dataset for training the VGG13 model, with the same preprocessing and augmentation as the best model described above. We repeated this procedure multiple times, making datasets with smaller images (Figure 2).

**Figure 3:**
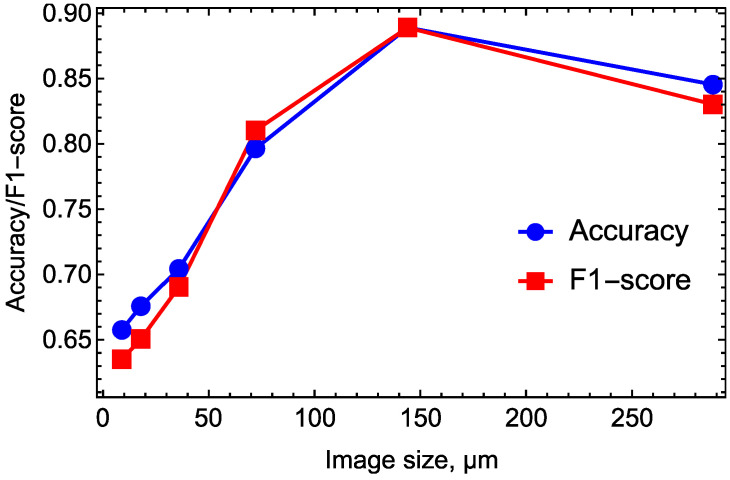
Two quality measures (accuracy and F1-score) shown on the validation set by the VGG13 model trained on images of different sizes. The maximum values are reached at ~144 µm.

The classification models trained on these datasets demonstrated varying performance, and the highest prediction quality on the validation set was observed for an image size of ~144 µm (Figure 3). Thus, this size can be interpreted as a characteristic scale on which the visual separation of the colony phenotypes occurs most effectively. This scale is intermediate between the typical sizes of cells and mature colonies, theoretically reflecting the fact that both the cellular and colonial morphological features should be taken into account when selecting the best colony [11]. Therefore, this scale should provide, at least partially, an estimate for the size of the colony sub-domains whose morphological changes are the most informative.

### 2.4. Proteome Analysis in H9 Cells with Good and Bad Phenotype

To reveal whether the H9 colonies with good and bad morphological phenotypes under self-renewal conditions differ at the molecular level, we analyzed the proteomic data of eight samples, consisting of good clonal undifferentiated colonies (good phenotype, 3 samples), clonal colonies with signs of spontaneous differentiation (bad phenotype, one sample), and non-clonal colonies with differentiated cells (bad phenotype, four samples; bulk cultures). The cell cultures for clonal and bulk expansion were propagated as described in Ref. [11].

**Figure 4:**
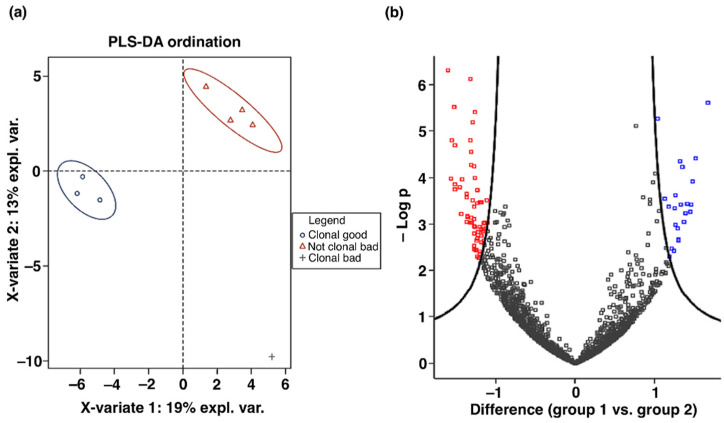
Proteome analysis in H9 cells with different phenotypes. (**a**) Three groups of H9 cells in the partial least squares discriminant (PLS-DA) analysis of the expressed proteins. The ellipses indicate the 95% confidence domains for each group. (**b**) Volcano plot showing statistically significant differences in the expression of proteins in experimental group 1 (good clonal cells) compared to group 2 (non-clonal cells with differentiation). Student’s T-test difference is shown on the horizontal axis, and minus logarithm of adjusted *p*-value on the vertical axis. Down-regulated proteins are highlighted in red, and up-regulated proteins are highlighted in blue.

A total of 1791 proteins were reliably identified in these samples of H9 cells. The ordination analysis showed a clear separation of the samples with different phenotypes based on the proteomic data (Figure 4a). We found 88 differentially expressed proteins, including 63 down-regulated and 25 up-regulated in experimental group 1 (good clonal cells) compared to group 2 (non-clonal cells with differentiation) (Figure 4b, Table S2).

## 3. Discussion

Cell reprogramming has allowed the generation of thousands of new hiPSC lines over the last decade. Currently, various molecular methods are used to assess the state of undifferentiated, “true” colonies of hiPSCs. However, the use of invasive methods of assessment does not allow for further application of these cells in clinical practice. In this regard, the morphological assessment of the colony as a non-invasive approach is a practical way for assessing their quality. We previously demonstrated that the use of morphological phenotype based on the phase-contrast image analysis correlated well with the clonality and pluripotency characteristics of three different hiPSC lines [11]. The existing various commercial high-content/high-throughput image acquisition systems, which are a useful tool in the study of other cell types, are not always applicable for the morphological assessment of hPSCs since multicellular colonies of the hPSCs are formed by close-packed, very small cells, many parameters of which may be “overlooked” by unspecific automatic image analysis [18,30,31]. These commercial image acquisition systems may “fail to notice” alterations in the cell’s morphology and therefore cannot guarantee their further safe use in the clinic. Development of the special to hiPSCs imaging platforms will undoubtedly help to transduce big-scale stem cell research into clinic.

Recently, several reports have described the development of automated systems for hiPSC generation and cultivation [7,32,33,34]. However, most of these systems focus on distinct cell culture steps, while comprehensive solutions covering all relevant processes are still scarce. Robotic high-throughput biomanufacturing and functional differentiation of hPSCs has recently been described [10]. The development of the StemCellFactory, a modular platform that automates the reprogramming process and enables the parallel derivation and expansion of hiPSC lines, will help to overcome several challenges, reduce the burden of manual hiPSC culture, and contribute to improving overall experimental reproducibility.

Developing tools for the automated control of hPSC cultivation attracts much attention and involves many different approaches [8,9,10,13]. In our study, we demonstrated the viability and practical usefulness of a straightforward approach, in which we first use an expert to phenotype H9 hESC colonies in a collection of images and then use deep learning methods to train an end-to-end classification model as a potential substitute for the expert. The advantage of this approach is that it does not require feature extraction as a prerequisite. It also relies on the fact that the morphological criteria in image analysis are sufficient to determine the phenotype and that they are consistent with the PCR data [11], so a morphology-based classification function is sufficient for use in an automatic image evaluation platform.

We found a specific combination of image preprocessing, augmentation, and CNN configuration that led to high phenotype prediction accuracy on validation images. This information may by specific only for the H9 line and the classification requirements considered in our study, but we believe that the general methods that we have considered should be useful for application to other cell lines and other class definitions. In particular, the VGG models proved to be among the most effective in image classification for various cellular systems [27].

The spontaneous differentiation of hPSC colonies associated with loosening pluripotency manifest themselves via several morphological changes, both at the cellular level and at the level of the whole colony. The cells change their shapes to less circular ones, with nuclear sub-domains becoming more variable [18]. The cells also tend to pack in a less compact fashion, leading to more intercellular space within the colony, while the colony perimeter becomes irregular [11]. All of these changes take place on different spatial scales, and it is hard to determine which scale is the most informative. Classification for the best clone recognizing using deep learning models allowed us to approach this question in a practical way. We showed that the image size of ~144 µm provided the most efficient visual separation of the colony phenotypes, at least for our cellular system.

Our additional proteome analysis of the H9 cells from the colonies that were used for the computational analysis revealed proteins differentially expressed in the groups of cells with distinct phenotype. This proteomic data clearly separated these groups, thus providing molecular evidence for a connection between the morphological changes and molecular markers in the differentiated cells. The presented result is a preliminary step towards a more comprehensive proteome analysis for hPSCs of various cell lines, which we are currently pursuing.

Our study has several limitations. We analyzed one cell line, thus reducing the probability that the classifier would be applicable for other lines. The specific medium that we used for hESC growth could also set constraints on the wider applicability of our results. The first defined media, mTESR1, described by Ludwig and colleagues in 2006 [35], are still one of the most widely used to grow hPSCs. The other, StemPro (Thermo Fisher Scientific, Waltham, Massachusetts, USA), is also used in combination with matrigel, vitronectin or laminin as the matrix. Later, Xeno-free, chemically defined media such as Essential 8 (E8) [36] and StemMacs iPS-Brew XF (Miltenyi Biotec, Surrey, UK) were developed. Currently, mTESR1 and E8 are regarded as the best for maintaining hPSCs and are routinely used in the research laboratories. At the same time, it should be emphasized that, at the beginning, with the appearance of the new culture medias, research has mainly focused on the questions related to the pluripotency maintenance; the morphological changes of cell cultures under different conditions have not been investigated. At present, it is known that the type of culture media impacts hPSC morphology and, in this way, may indicate the preferential lineage choice for further differentiation [37]. Interestingly, the StemPro and mTESR1 media demonstrated equivalent lineage differentiation propensity, however undefined. The conditional media showed increased differentiation towards mesoderm and ectoderm [37]. It should be noted that the morphological responses of the various hPSC lines under different growth conditions have not been sufficiently studied. We plan to devote our further attention to this issue and explore the possibility of adapting our classifier to different lines under various culture conditions.

Despite these limitations, we believe that our results are a good preliminary step towards the development of a truly automated instrument for hPSC quality control based on an end-to-end classification approach. In the future, it will be possible to move to the creation of a computer software for the automatic recognition of the best pluripotent clones when working with a large volume of cell cultures, which will make this process more efficient, reliable, and economical. With the incorporation of modern computer technology and knowledge of stem cell biology, an increased demand and introduction of automated platforms for stem cell research is expected, which will improve the efficiency and reliability of the use of these cells in clinical practice.

## 4. Materials and Methods

### 4.1. Cell Culture, Image Acquisition, and Colony Phenotyping

The human embryonic stem cell line H9 (WiCell, Madison, WI, USA) was passaged on 6-well plates coated with hESC-qualified Matrigel Matrix (Corning Matrigel Matrix, Life Sciences, NY, USA), manually or via bulk expansion, at a 1:4 split ratio using 0.02% EDTA (Versene) dissociation solution and 10 µM ROCK inhibitor (Y-27632; StemCell Technologies, Cambridge, UK). A volume of 2 mL of mTERSR1 medium (StemCell Technologies) was used per well. The cell culture was checked daily. During manual colony propagation, small cell clumps, of 15–20 cells per clamp, were used from the colony for clonal expansion. The culture was kept under standard condition for 5 days at 37 °C with 5% CO_2_ atmosphere and 21% O_2_ according to WiCell Inc. protocols.

Phase-contrast images were taken of 269 colonies of the middle passage (p36, 96–120 h) with a resolution of 1280 × 960 pixels (290 × 218 µm^2^/image). For the imaging analysis, colonies were selected from seven different independent platings, represented by different freezing stocks into different wells. The colonies from the images were visually analyzed and phenotyped as “good” or “bad”, depending on the morphological properties associated with the potential loss of pluripotency, as described before [11]. As our aim was to develop an automatic system for the analysis of large volumes of cultures in stem cell banks, we drew a line separating the colonies at the middle state, where the colonies have some good cells together with differentiated ones, as even a part of the colony showing signs of differentiation must be removed by the operator, mechanically. When working with a large volume of cell culture in stem cell banks, the delicate work required to remove the differentiated part becomes inefficient and impossible. For that reason, in the current study, we assigned the colonies at the middle stage of spontaneous differentiation to the bad phenotype, thus keeping only two phenotypes for classification. One of the authors, Dr. Irina Neganova, was responsible for the phenotyping. She has been working with hPSCs since 2006, has published 38 articles on the subject and thus is a highly competent specialist in this field.

In brief, the colonies were assigned a good phenotype if they showed no signs of spontaneous differentiation, which was morphologically expressed via a flat structure, prominent well-defined edge, and a high nuclear-to-cytoplasmic ratio, with prominent nucleoli in square-shaped, tightly packed cells. The colonies with the assigned bad phenotype possessed loosely packed cells with phase-bright gaps visible between cells, with altered cell morphology (elongated cells) and greatly varying cell sizes. In addition, the “spiky” colony edges were associated with the bad colony phenotype. There were 137 (51%) good and 132 (49%) bad colonies in the collected images.

### 4.2. Image Preprocessing and Augmentation

To reduce the number of training parameters, the raw images were proportionally compressed to a size of 256 × 256 pixels. The following image preprocessing methods were considered: gray level transformation, intensity normalization, binarization, and histogram equalization. Gray level transformation converts the image to grayscale, with the new pixel intensity Y (0 ≤ Y ≤ 255) equal to Y = 0.299R + 0.587G + 0.114B, where R, G, and B are intensities in the red, green, and blue channels, respectively. Intensity normalization is the min–max scaling, reducing pixel intensities to the values between 0 and 1. Binarization transforms an image to black and white, setting the pixel intensity to either 0 or 1 depending on its relation to a fixed threshold. Histogram equalization is an image processing technique used to enhance contrast. The intensity distribution in the image is transformed in such a way that the intensity histogram is stretched to the unit interval of modified intensity values.

The image augmentation methods included random cropping, when a sub-domain of the image was randomly selected as a new image in the dataset, and random rotations. The random rotations included rotations of the original image by an angle multiple of 90°, vertical and horizontal flipping, and transposition.

### 4.3. CNN Model Selection and Training

The VGG network was taken as a basis for constructing a CNN for phenotype classification problem [29]. Five architectures (VGG13, VGG13–FirstPool4, VGG12, VGG12–FirstPool4, and Res + VGG13) were specifically considered and described in detail in Table S1. All types of CNN were programmed with the help of PyTorch machine learning framework for Python programming language, version 3.8 (https://pytorch.org/, accessed on 15 November 2022). The dataset was split into training and validation parts in a 4:1 ratio. The following binary cross-entropy loss function was minimized during the training: $L=-1/N\sum_{i=1}^{N}y_{i}\log{\hat{y}}_{i}+\left(1-y_{i}\right)\log\left(1-{\hat{y}}_{i}\right)$, where $y_{i}$ is the observed class label for *i*th image (1 for good and 0 for bad phenotype), ${\hat{y}}_{i}$ is the predicted probability that the image contains a good colony (output of the model), and *N* is the number of images in the dataset.

The conventional prediction quality measures of the model were calculated on the validation set. Accuracy represents the ratio of correctly classified samples to the total number of samples in the dataset. Precision is the ratio of true positive predictions to the sum of true positives and false positives. Recall, also known as the true positive rate or sensitivity, is the ratio of true positive predictions to the sum of true positives and false negatives. F1-score is the harmonic mean between precision and recall, which means that it penalizes the maximum values of either: F1 = 2 × precision × recall/(precision + recall). AUC stands for the Area Under the ROC Curve and describes a cumulative measure of model performance across all possible classification thresholds. AUC values range between 0 and 1: AUC = 0 for a model whose predictions are all wrong, and AUC = 1 if all predictions are correct.

### 4.4. Proteome Analysis

For the proteome analysis of the hESC H9 colonies with good and bad morphological phenotype, the colonies were selected by the operator, according to the morphological criteria that were described in detail and justified in Ref. [11]. It is important to note that the colonies were selected in the same way as for image analysis, i.e., from different wells of various stocks platings. The proteome analysis was carried out using equipment of the “Human Proteome” Core Facility Center (Institute of Biomedical Chemistry, Moscow, Russia). The H9 cell samples were lysed using ice-cold buffer (150 µL) containing 5% SDS with subsequent ultrasonication using the Bandelin Sonopuls probe (“BANDELIN electronic GmbH and Co. KG”, Berlin, Germany). The sample protein concentration was measured using a Pierce™ BCA Protein Assay Kit (Pierce, Rockford, IL, USA). Trypsin digestion was then performed according to the S-Trap sample preparation method [38]. Then, peptides (100 μg in 100 μL) of 8 samples were processed with a 10-plex TMT kit (Thermo Fisher Scientific, Rockford, IL, USA) based on provided recommendation. The HPLC-MS/MS analysis of obtained peptides was performed using an Ultimate 3000 RSLCnano chromatographic HPLC system (Thermo Scientific, Rockford, IL, USA) connected to a mass spectrometer Q-Exactive HFX (Thermo Scientific, Rockford, IL, USA). Procedures for HPLC-MS/MS, protein identification, and TMT-based quantitation were described previously [39].

The statistical analysis was performed in the Perseus software, version 1.6.15.0 [40], with the loading of «NormRIC» values. The protein groups which are known potential contaminants, only identified by site or reverse, were removed. Only proteins with two or more peptides detected were included in the analysis. The data was logarithmized (log2(x)) for further analysis, and z-score normalization was applied. The protein groups not reaching 90% valid values of the “NormRIC”, at least in one experimental group, were filtered out. The imputation of the remaining missing values was performed based on sampling from a normal distribution with default Perseus’s parameters. The differential protein abundances between experimental groups were tested using t-tests with correction for multiple testing (permutation-based FDR), considering *q*-value < 0.05 and multiplicity of change FC > 2 as significant. The ordination of the samples by Principal Component Analysis (PCA) and sparse Partial Least Squares Discriminant Analysis (sPLS-DA) was performed in the package “MixOmics” [41] using R version 4.1.2 (https://www.R-project.org/, accessed on 15 November 2022).

## Figures and Tables

**Table 1:** Models with various CNN architectures and their measures of classification quality on the validation set. Best values are highlighted in bold. No processing or augmentation was used on the input images. Quality measures are defined in Methods. More details about network architectures are given in Table S1.

Model Configuration	Quality Measures				
	Accuracy	Precision	Recall	F1-Score	AUC
VGG13	**0.83**	0.85	**0.81**	**0.83**	**0.99**
VGG13–FirstPool4	0.80	0.88	0.74	0.81	0.99
VGG12	0.74	0.81	0.70	0.75	0.98
VGG12–FirstPool4	0.69	**0.92**	0.62	0.74	0.95
Res + VGG13	0.80	0.85	0.76	0.80	0.98

**Table 2:** Classification quality measures on the validation set for the VGG13 model with various preprocessing methods applied to the input images. Best values are highlighted in bold. Quality measures and image preprocessing methods are described in Methods.

Preprocessing Method	Quality Measures				
	Accuracy	Precision	Recall	F1-Score	AUC
no preprocessing	0.83	0.85	**0.81**	0.83	0.99
gray level transform	0.80	0.92	0.73	0.81	0.99
binarization	0.70	0.93	0.63	0.76	0.98
normalization	0.80	0.85	0.76	0.80	0.99
histogram equalization	**0.84**	**0.93**	0.77	**0.84**	**0.99**

**Table 3:** Classification quality measures on the validation set for the VGG13 model with various augmentation methods applied to the input images preprocessed with histogram equalization. Best values are highlighted in bold. Quality measures and image augmentation methods are described in Methods.

Augmentation Method	Quality Measures				
	Accuracy	Precision	Recall	F1-Score	AUC
no augmentation	0.84	0.93	0.77	0.84	0.99
rotations	0.85	0.85	0.85	0.85	0.98
cropping	0.85	0.92	0.80	0.86	0.99
rotations + cropping	**0.89**	**0.93**	**0.86**	**0.89**	**0.99**

**Table 4:** Confusion matrix on the validation dataset (*n* = 54) for the best accuracy model from Table 3.

	Predicted: Good	Predicted: Bad
Actual: Good	24	2
Actual: Bad	4	24

## Data Availability

The dataset with images and code can be found at the Zenodo repository (https://doi.org/10.5281/zenodo.7316404 (accessed on 13 November 2022)).

## Supplementary Materials

The following supporting information can be downloaded at: https://www.mdpi.com/article/10.3390/ijms24010140/s1, Table S1: Architectures (sequence of layers) of CNNs considered in the study. Table S2: List of differentially expressed proteins. Figure S1: Characteristic morphological portraits of hESC H9 colonies with good and bad phenotypes.
