# Supplementary material for: Quality Control of Human Pluripotent Stem Cell Colonies by Computational Image Analysis Using Convolutional Neural Networks
Source: Int J Mol Sci. 2022 Dec 21;24(1):140. doi: 10.3390/ijms24010140 (PMC9820636; doi:10.3390/ijms24010140)
Supplement: Supplementary file 1 [file ijms-24-00140-s001.zip › Figure S1.pdf]

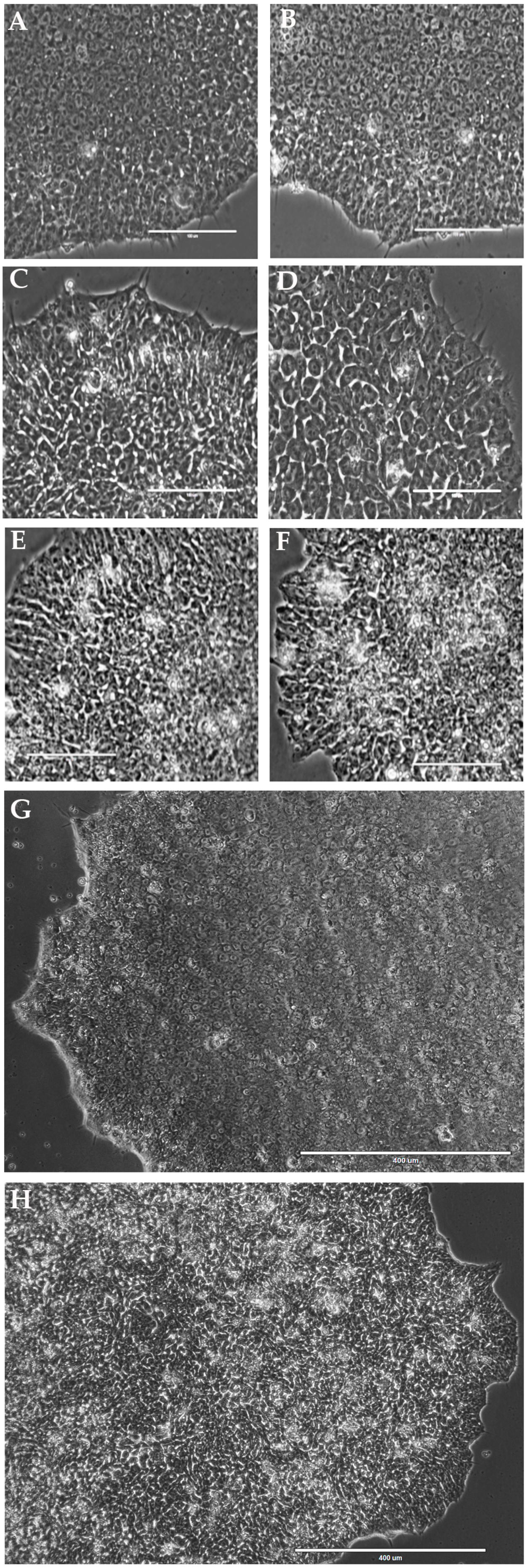

Figure S1. Characteristic morphological portraits of hESC H9 colonies with good and bad phenotypes. **A:** Colony with good phenotype: colony with densely packed cells and a well-defined edge. All cells are about the same size. Along the edge of the colony, the cells are also rounded. The nuclei are well distinguished. **B:** Colony of a good phenotype. Note that some elongated cells appeared at the periphery. **C:** Colony of a bad phenotype – cells are sparse and differ in size. **D:** Colony with a bad phenotype. Cells became much larger and loosely arranged. **E–F**: Along the edge of the colonies, a belt with elongated cells is clearly visible. Cells are located randomly towards the center of the colonies. It is noticeable that many cells are detached from the colony. **A–F:** 96h in culture; scale – 100 µm. **G:** Colony of a good phenotype. Note that the colony is flat; cells throughout the colony are of equal size and densely packed. Along the edge of the colony, the cells are also densely packed and have a rounded shape. **H:** Colony of a bad phenotype. Cells are packed randomly with lots of free space between them; along the edge of the colony, a belt of the elongated cells is clearly visible. **G–H:** 120h in culture; scale – 400 µm.
