# Supplementary material for: Quality Control of Human Pluripotent Stem Cell Colonies by Computational Image Analysis Using Convolutional Neural Networks
Source: Int J Mol Sci. 2022 Dec 21;24(1):140. doi: 10.3390/ijms24010140 (PMC9820636; doi:10.3390/ijms24010140)
Supplement: Supplementary file 1 [file ijms-24-00140-s001.zip › Table S1.pdf]

Table S1. Architectures (sequence of layers) of CNNs considered in the study. 'ConvN1-N2' denotes a convolutional layer with a kernel of size N1xN2; 'maxpool N1 x N2', max-pooling layer with a N1xN2 filter; 'FC-N', fully connected layer with N nodes; 'skip connection', connection that skips one or more layers; it performs an identity mapping and is added to the output of the convolutional layer; 'sigmoid', sigmoid activation function for a binary classification.

CNN configuration				
VGG13	VGG13 FirstPool4	VGG12	VGG12 FirstPool4	Res+VGG13
input: 256 x 256 RGB image				
conv3-16<br>conv3-16	conv3-16<br>conv3-16	conv3-16<br>conv3-16	conv3-16<br>conv3-16	conv3-16<br>conv3-16<br>skip connection
maxpool 2 x 2	maxpool 4 x 4	maxpool 2 x 2	maxpool 4 x 4	maxpool 2 x 2
conv3-32<br>conv3-32	conv3-32<br>conv3-32	conv3-32<br>conv3-32	conv3-32<br>conv3-32	conv3-32<br>conv3-32<br>skip connection
maxpool 2 x 2				
conv3-64<br>conv3-64	conv3-64<br>conv3-64	conv3-64<br>conv3-64	conv3-64<br>conv3-64	conv3-64<br>conv3-64<br>skip connection
maxpool 2 x 2				
conv3-128<br>conv3-128	conv3-128<br>conv3-128	conv3-128<br>conv3-128	conv3-128<br>conv3-128	conv3-128<br>conv3-128<br>skip connection
maxpool 2 x 2				
conv3-128<br>conv3-128	conv3-128<br>conv3-128	conv3-128<br>conv3-128	conv3-128<br>conv3-128	conv3-128<br>conv3-128<br>skip connection
maxpool 2 x 2				
conv3-128<br>conv3-128	conv3-128<br>conv3-128	conv3-128	conv3-128	conv3-128<br>conv3-128<br>skip connection
maxpool 2 x 2				
FC-512	FC-128	FC-512	FC-128	FC-512
output: sigmoid for classification probability				
